# Clinical outcomes of avelumab and pembrolizumab in advanced urothelial cancer: an observational multicenter retro-prospective study on patients undergoing treatment in clinical practice (AVePEm study)

**DOI:** 10.3389/fonc.2025.1532421

**Published:** 2025-02-21

**Authors:** Francesca Zacchi, Anna Sordo, Irene Torresan, Clara Lorenzi, Ester Tasselli, Sarah Pafumi, Silvia Palmerio, Emilia Durante, Nicola Inzerilli, Mattia Pellicciari, Davide Pastorelli, Michele Milella, Giulia Lorenzoni, Sara Merler, Andrea Zivi

**Affiliations:** ^1^ Section of Innovation Biomedicine-Oncology Area, Department of Engineering for Innovation Medicine (DIMI), University of Verona and University and Hospital Trust (AOUI) of Verona, Verona, Italy; ^2^ Centro Ricerche Cliniche, Azienda Ospedaliera Integrata, Verona, Italy; ^3^ Department of Medicine, University of Verona, Verona, Italy; ^4^ Unit of Biostatistics, Epidemiology and Public Health, Department of Cardiac, Thoracic, Vascular Sciences and Public Health, University of Padova, Padova, Italy; ^5^ Department of Oncology, AULSS 9 Scaligera, Verona, Italy; ^6^ Oncology, Pederzoli Hospital, Peschiera del Garda, Italy

**Keywords:** urothelial cancer, immunotherapy, avelumab, pembrolizumab, survival

## Abstract

**Introduction and objectives:**

Patients (pts) with metastatic urothelial carcinoma (mUC) gain substantial benefit from immunotherapy exposure. If they do not experience disease progression after 4-6 cycles of first-line platinum-based chemotherapy (PBC), they may benefit from immunotherapy as maintenance treatment with Avelumab; otherwise, Pembrolizumab is an approved second-line therapy after disease progression on first-line chemotherapy. However, no clinical trial data currently demonstrate which treatment strategy offers superior survival outcomes.

**Patients and methods:**

This is a multicenter, observational, retro-prospective study involving pts with mUC who did not progress after 4-6 cycles of PBC: GroupA received Avelumab and GroupB Pembrolizumab. The primary endpoints were overall survival (OS) and progression-free survival (PFS), with neutrophil-to-lymphocyte ratio (NLR) ≥3 at the baseline of PBC and at the start of immunotherapy in predicting outcome, adverse events (AEs), subsequent therapies after the immunotherapy strategy, and costs associated with these treatments as secondary endpoints.

**Results:**

From August 2019 to October 2024, we identified 30 pts. Of those, 53% were in GroupA and 47% in GroupB. The mOS in GroupA was 27 mo and in GroupB 26 mo and the mPFS of immunotherapy was 7.5 mo and 5.5 mo. At the time of data analysis, 33% (n=10) of pts were alive and 27% (n=8) on treatment, with 38% (n=3) still receiving Avelumab, and 50% (n=4) and 12% (n=1) on subsequent therapies after Avelumab and Pembrolizumab, respectively. Approximately 55% of patients in both groups had a baseline neutrophil-to-lymphocyte ratio (NLR) ≥3 at the baseline of PBC. No statistically significant association was found between NLR, whether considered as a continuous or dichotomous variable, and overall survival or progression free survival. Both treatments were well tolerated, with Grade 3 AEs in 1 pt on Avelumab and 3 on Pembrolizumab, and no Grade 4 AEs reported.

**Conclusions:**

The two immunotherapy strategies showed no significant differences in OS and PFS. Of note, more pts were on Avelumab treatment at the data cut-off. AEs were similar in the two groups. Further investigation and follow-up are warranted to gain definitive conclusions on optimal mUC management in the era of immunotherapy and immunoconjugates.

## Introduction

Bladder cancer is the second most common urological tumor following prostate cancer, ninth in incidence and thirteenth in mortality worldwide, based on the latest GLOBOCAN data (1). Transitional cell carcinoma or urothelial carcinoma (UC) is the most frequent histotype, representing 90% of cases (2). The whole urinary tract can be affected by this disease. Upper urinary tract tumors (UTUC) represent 5-10% of UC, with an annual incidence of 2 cases for 100000 population in Western Countries (3). Patients affected by advanced urothelial carcinoma (aUC) have a poor prognosis in the absence of active treatment with a median survival of 3-6 months (4–7).

Starting from the mid-20th century, platinum-based chemotherapy has been the cornerstone for the treatment of aUC. Chemotherapy regimens MVAC (methotrexate/vinblastine/doxorubicin/cisplatin) or cisplatin plus gemcitabine have demonstrated a median Overall Survival (mOS) around 1 year (12-15 months) and a median Progression Free Survival (mPFS) of 6-8 months (6, 7). The possibility to combine carboplatin and gemcitabine, according to the phase II/III study EORTC 30986, for cisplatin-ineligible patients led to the achievement of a mOS of 9.3 months and a mPFS of 5.8 months (8). Upon failure of first-line therapy, taxane-based chemotherapy or vinflunine have historically been offered, with a mOS of 6-7 months (9, 10).

The advent of immune checkpoint inhibitors (ICIs) has implemented and revolutionized therapeutic strategies for aUC. Since 2017, the second-line setting has benefitted from the introduction of ICIs, with Pembrolizumab. KEYNOTE-045 study evaluated patients with aUC who developed disease recurrence or progression following first-line platinum-based treatment. The study demonstrated a mOS of 10.3 months with Pembrolizumab (95% CI, 8.0 to 11.8) compared with 7.4 months (95% CI, 6.1 to 8.3) in the standard chemotherapy group (taxanes or vinflunine at the investigator’s choice), but no significant increase in mPFS, in the ITT population. The benefit of Pembrolizumab over second-line chemotherapy occurred in all subgroups examined in the study, including patients with liver metastasis and patients with PD-L1 CPS < 1% (11). The 5-year update of the study showed an interesting finding regarding the duration of response (DOR): the median DOR was 29.7 months for Pembrolizumab compared with 4.4 months for chemotherapy (12).

From 2020, patients with aUC in response or disease stability to first-line chemotherapy (4-6 cycles of cisplatin or carboplatin in combination with gemcitabine) may benefit from the addition of maintenance Avelumab, based on the results of the JAVELIN Bladder 100 phase 3 study. In Avelumab-treated patients, mOS and mPFS were 21.4 months (95% CI, 18.9 to 26.1) and 3.7 months (95% CI, 3.5 to 5.5), respectively, in the overall study population, and not reached (95% CI, 20.3-NE) and 5.7 months (95% CI 3.7 to 7.4) in the PDL1-positive patient population (13). The long-term results of the study, at a follow-up longer than 38 months, demonstrated a 2-year survival rate of 49.8% in the Avelumab arm compared with 38.4% in the BSC group (14). At the pre-specified subgroup analysis, the benefit of Avelumab was confirmed in both cisplatin- and carboplatin-treated patients, regardless of PD-L1 expression, and was comparable in patients in response or disease stability to first-line chemotherapy. Visceral disease within liver or lung resulted in a lower mOS and mPFS in the *post-hoc* subgroup analysis (15).

Over the years, many factors have been explored trying to find prognostic or predictive factors to systemic therapy. Firstly, Karnofsky Performance Status (PS) and disease sites were found to be prognostic - patients with lymph nodes-only disease have a 5-year OS of 20.9% compared to 6.8% in patients with visceral involvement at the diagnosis (2, 7). Then, the Bellmunt risk score allowed stratification of aUC patients refractory to platinum-based therapy into 4 groups with different outcomes in OS, based on the following pre-treatment parameters: Eastern Cooperative Oncology Group (ECOG) PS, haemoglobin levels, and presence of liver metastasis (16). The Bellmunt score was then implemented for patients initiating a second-line therapy with ICIs, showing that the addition of the C-reactive protein parameter improved stratification and prognostic accuracy for aUC patients treated with ICIs (17). Moreover, Bamias et al. proposed a different prognostic model for patients with aUC and treated with ICIs: in the 936 patients analysed, ECOG PS, alkaline phosphatase values, neutrophil/lymphocyte ratio, presence of liver or bone metastasis, and time since last chemotherapy infusion were independent prognostic factors (18). Prior studies have also reported that certain systemic inflammation markers calculated using peripheral blood count values (such as the neutrophil to lymphocites ratio - NLR) were shown to be prognostic for patients with urothelial carcinoma and other solid malignancies (19, 20).

Given this background, patients with advanced urothelial carcinoma (aUC) who do not experience disease progression after platinum-based first-line chemotherapy may benefit from two different immunotherapy options: maintenance Avelumab therapy following first-line treatment (13) or Pembrolizumab as a second-line option after disease progression (11).

When immunotherapy data are taken together, there is no definitive evidence of superiority in mOS between the two above-described strategies of therapy. Therefore, the optimal sequence and timing for using immune checkpoint inhibitors (ICIs) remains unclear. Additionally, the KEYNOTE-045 and JAVELIN Bladder 100 trials involved highly selected patient populations, making their findings less generalizable to the broader patient population.

Our multicenter retrospective study wanted to evaluate the outcomes, tolerability, and costs of Avelumab as first-line maintenance therapy versus Pembrolizumab as second-line therapy in patients with aUC who have not progressed on platinum-based first-line chemotherapy. The analysis aims to provide crucial information on the real-world effectiveness of these therapies, optimize therapeutic strategies, and explore potential predictive or prognostic biomarkers, particularly considering the latest emerging treatments for advanced urothelial cancer.

## Patients and methods

AVePEm is a multicenter, observational, retro-prospective study conducted across three Italian referral centers. Ethical approval for the study was granted by the Verona Ethical Committee on August 2, 2024 (Approval Code: 339CET). The data cut-off for analysis was October 31, 2024.

**Figure 1:**
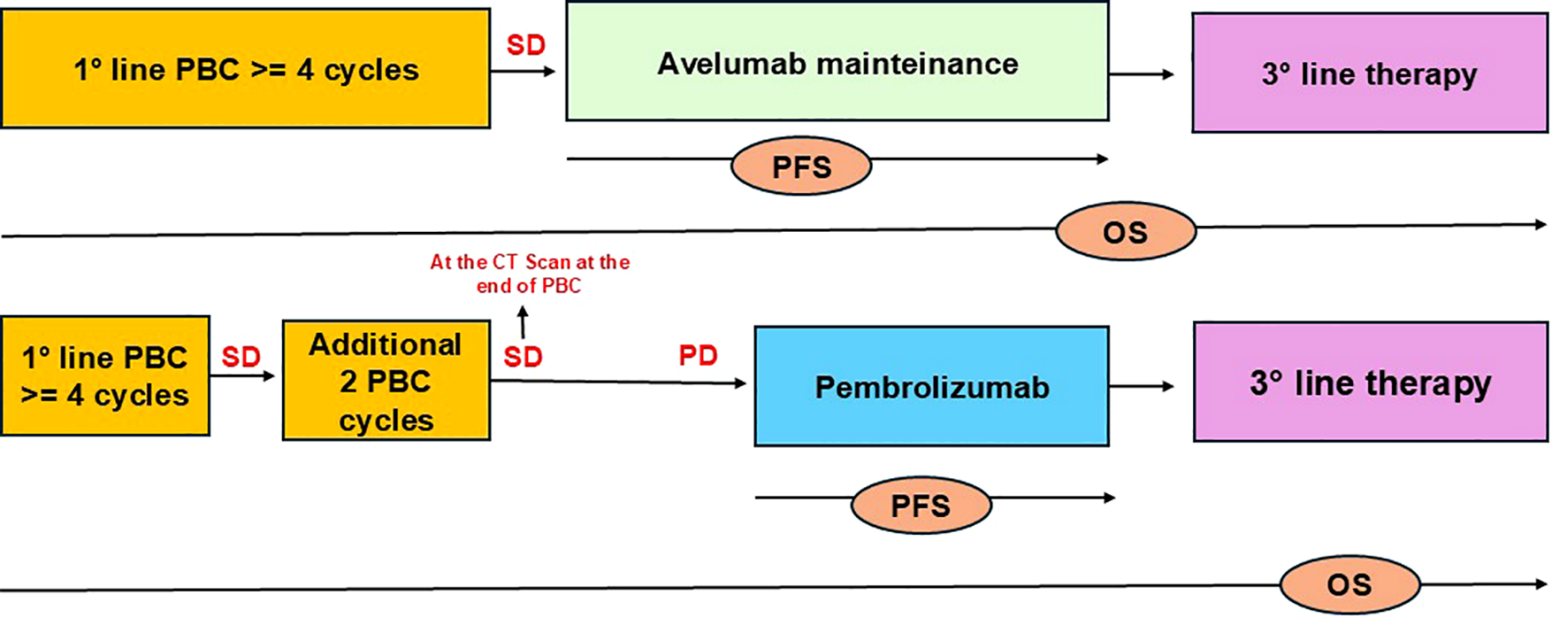
Flowchart illustrating the patient selection. The study included patients with mUC who did not progress after 4-6 cycles of PBC: GroupA received Avelumab and GroupB Pembrolizumab. OS was defined as the time from treatment initiation (first-line platinum chemotherapy) until death, while PFS was defined as the time from the start of immunotherapy until death, radiographic or clinical progression to immunotherapy (Avelumab or Pembrolizumab).

The study involves patients with platinum-fit mUC who did not experience disease progression following 4-6 cycles of first-line platinum-based chemotherapy. The analysis includes two groups: patients receiving Avelumab as first-line maintenance therapy (Group A) and patients who achieved stable disease (SD) or partial response (PR) after 4-6 cycles of platinum-based chemotherapy, followed by Pembrolizumab as second-line therapy after disease progression (Group B) (**Figure 1**).

In Group B, no patients experienced disease progression after 4–6 cycles of PBC, making this population comparable to those receiving Avelumab as maintenance therapy per standard of care. Drugs were administered as per label, in second-line (pembrolizumab) or in first-line maintenance (avelumab) for metastatic disease. Clinical data were extracted from electronic patient records. At the time of their first visit to our institution, all patients consented to the use of their clinical data for scientific purposes.

To be eligible for the study patients must present with a good performance status (ECOG PS 0-2) and a pathologically confirmed diagnosis of stage IV urothelial carcinoma, regardless of PD-L1 status.

The study’s primary endpoints were OS and PFS for both groups of patients who received Avelumab (GroupA) or Pembrolizumab (GroupB) after platinum-based chemotherapy, while secondary endpoints included adverse events (AEs), subsequent therapies after Avelumab and Pembrolizumab, as well as the costs associated with these immunotherapies.

A p-value of <0.05 was considered statistically significant.

Data collection included patient demographics (age, sex), tumor characteristics and treatment history (e.g., prior cystectomy, systemic therapy, baseline complete blood counts). Response evaluation for first-line chemotherapy and immunotherapy was based on RECIST version 1.1, with patients classified as responders (those with SD, PR, or complete response [CR]) or non-responders (those with progressive disease [PD]) according to iRECIST criteria.

OS was defined as the time from treatment initiation (first-line platinum chemotherapy) until death, while PFS was defined as the time from the start of immunotherapy until death, radiographic or clinical progression to immunotherapy (Avelumab or Pembrolizumab).

The neutrophil-to-lymphocyte ratio (NLR) was calculated at baseline prior to the start of chemotherapy and at baseline of the start of Immunotherapy (Avelumab or Pembrolizumab). The NLR was calculated by the absolute neutrophil count (number of cells × 10^3^/μL) divided by the absolute lymphocyte count (number of cells × 10^3^/μL). Both continuous and categorized (according to a cut-off of 3) NLR were considered in the analyses. The NLR cut-off value of 3 was chosen based on previous studies in the literature, which have shown that NLR of ≥3 or ≥3.65 in metastatic urothelial cancer are associated with worse PFS and OS outcomes (19, 21).

Adverse events were graded according to the Common Terminology Criteria for Adverse Events (CTCAE) v5.0.

Clinical data were extracted from electronic patient records. At the time of their first visit to our institution, all patients consented to the use of their clinical data for scientific purposes.

### Statistical analysis

Descriptive statistics were reported as median (I quartile – III quartile) for continuous variables and absolute numbers (percentages) for categorical variables.

Survival distribution at follow-up was evaluated using the Kaplan-Meier method, while cumulative incidence functions (CIFs) were employed for disease progression to account for competing risks.

Univariable Cox regression models were employed to evaluate the association of baseline characteristics of interest with overall survival (OS) and progression-free survival (PFS). Results were reported as Hazard Ratio (HR), 95% Confidence Interval (CI), and p-value.

A p-value <0.05 was considered statistically significant. Analyses were performed using the R software.

## Results

### Patient characteristics

From August 2019 to October 2024, 30 patients were identified to satisfy the required study inclusion criteria. 16 (53%) patients were treated with Avelumab as 1L maintenance (Group A), whereas 14 (47%) started Pembrolizumab as 2L therapy at disease progression (Group B).

The median age at diagnosis was 70.5 years (range 47-80), and 83% were male. The large majority (83%, n=25) were represented by urothelial carcinoma, 77% from lower and 23% from upper urinary tract. Among other histotypes, 2 had a mixed squamous - urothelial carcinoma of the bladder diverticulum, and 3 had a variant urothelial carcinoma only (2 squamous cell carcinoma, one originating from the renal pelvis and one from urethra, and 1 poorly differentiated carcinoma with sarcomatoid and rhabdoid features of the bladder). At diagnosis, 14 pts (47%) have lymph nodes-only disease, and 16 pts (53%) have visceral sites of disease (56% lung, 12.5% liver and 37.5% other sites). The median number of chemotherapy cycles was 6, 40% were ineligible for cisplatin and received carboplatin. At the start of chemotherapy, 70% of patients had a PS 0 ECOG and 30% a PS 1 ECOG.

Patients’ demographic data and principal clinical characteristics are summarized in **Table 1**.

**Table 1:** Patients characteristics.

Characteristic	Number of patients (n=30)
Gender	
Male	25 (83%)
Female	5 (17%)
Age at diagnosis	
Median age (range) - yr	70.5 (47-81)
Age ≥ 75 yr - no (%)	9 (30%)
ECOG Performance Status	
0	21 (70%)
1	9 (30%)
Primary site of origin	
Upper tract	7 (23%)
Lower tract	23 (77%)
Histologic variant	
Urothelial carcinoma	25 (83%)
Urothelial carcinoma, mixed type	2 (7%)
Variant urothelial carcinoma only	3 (10%)
Surgery on primary tumor	
Yes	19 (63%)
No	11 (37%)
Site of metastasis	
Lymph node only	14 (47%)
Bone	4 (13%)
Visceral site	16 (53%)
Lung	9 (56%)
Liver	2 (12.5%)
Other	6 (37.5%)
CDDP elegibility status	
Eligible	18 (60%)
Ineligible	12 (40%)
Type and cycles of 1L PBC	
CDDP/gemcitabine x 6	11 (37%)
CDDP/gemcitabine x 4	7 (23%)
CBDCA/gemcitabine x 6	11 (37%)
CBDCA/gemcitabine x 4	1 (3%)
BOR to 1L PBC	
PR	25 (83%)
SD	5 (17%)
ICI setting	
Avelumab maintenance	16 (53%)
Pembrolizumab 2L	14 (47%
NLR at CT start	
Low (< 3)	11 (37%)
High (≥ 3)	17 (57%)
NA	2 (7%)
NLR at ICI start	
Low (< 3)	15 (50%)
High (≥ 3)	13 (43%)
NA	2 (7%)
Radiotherapy for oligoPD on ICI	
Yes	5 (16%)
No	25 (83%)

### Outcomes: PFS, OS

**Figure 2:**
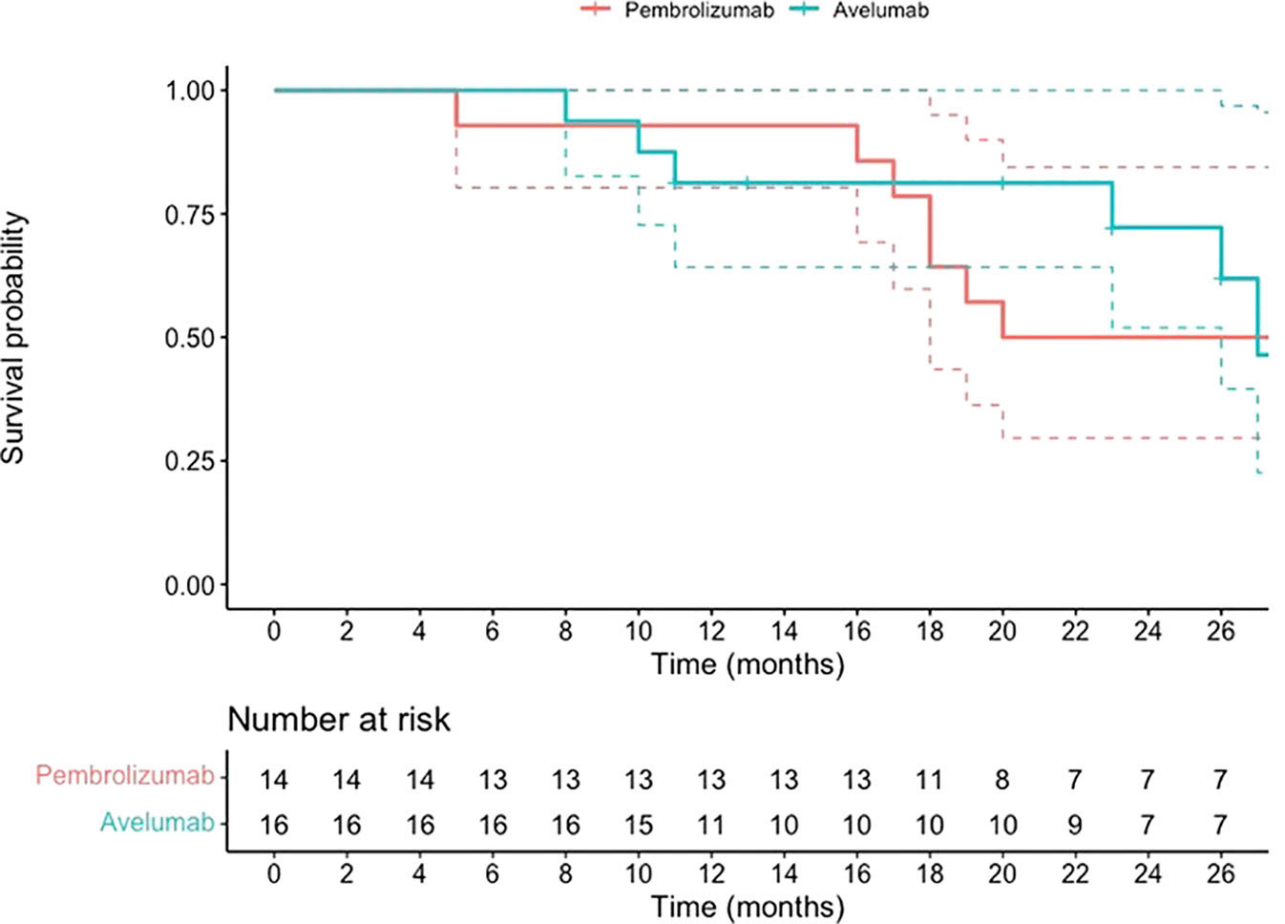
OS data for patients with mUC who began maintenance treatment with Avelumab following platinum-based chemotherapy or received Pembrolizumab as a second-line therapy after disease progression on first-line platinum-based chemotherapy.

The Kaplan-Meier survival curve, presented in **Figure 2**, shows a 24-month survival probability of 72% in both groups, with no significant differences (HR 0.95, 95% CI 0.34-2.61, p-value 0.92). The median survival in the Avelumab-treated group was 27, while in the Pembrolizumab-treated group was 26.

**Figure 3:**
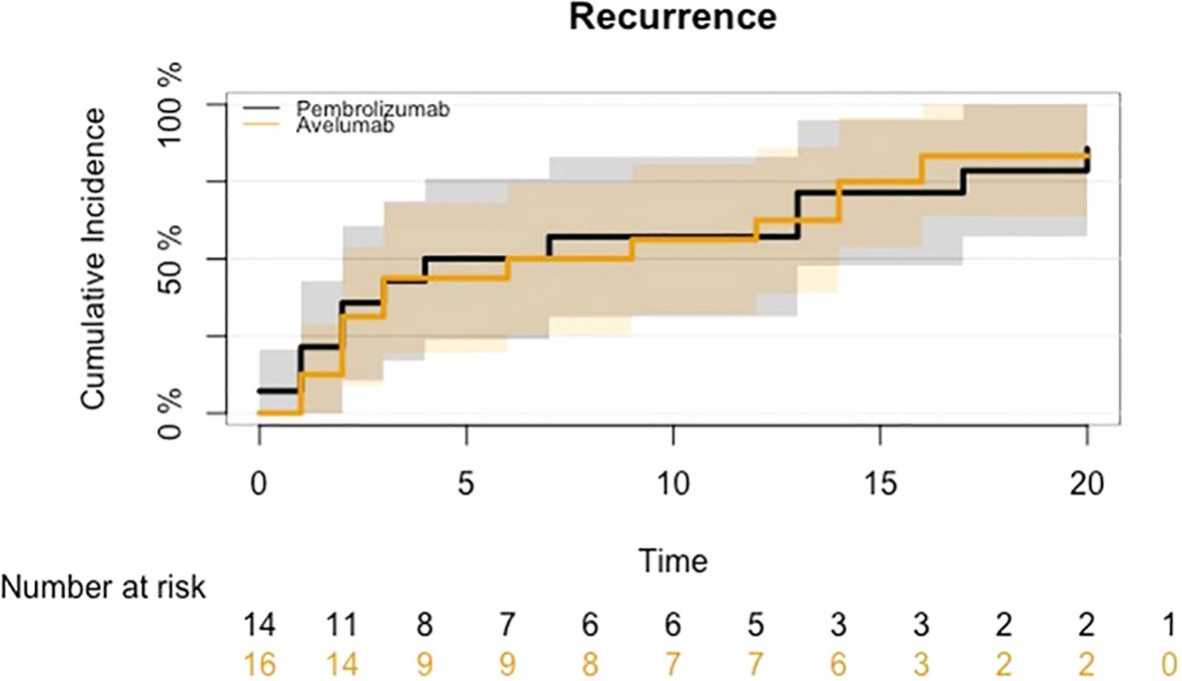
CIF for disease progression in mUC who began maintenance treatment with Avelumab following platinum-based chemotherapy or received Pembrolizumab as a second-line therapy after disease progression on first-line platinum-based chemotherapy.

The cumulative incidence of disease progression is presented in **Figure 3**; no significant differences were detected (HR: 0.88, 95% CI 0.40-1.94; p-value 0.75).

The best overall response (BOR) to 1L PBC, according to RECIST 1.1, was PR in 88% of patients (n=14) and SD in 12% of patients (n=2) for GroupA and PR 78.5% (n=11) and 21.5% SD (n= 3) for GroupB. Notably, the median interval between the end of first-line platinum-based chemotherapy (PBC) and the initiation of Pembrolizumab was 4.1 months (range 1-20 months). For Avelumab, this median time interval was 1.3 months (range 0.4-2.2 months).

The median duration of treatment was 7.5 months (range 1.6 - NR months) for Avelumab and 3.5 months (range 0 - 33 months) for Pembrolizumab, with a median number of 15 cycles (range 4-NR) and 5 cycles (range 1-38) administered, respectively.

The BOR to Avelumab according to RECIST 1.1 was 13% CR (n=2), 19% PR (n=3), 31% SD (n=5) and 37% PD (n=6). For Pembrolizumab, the BOR was 7% PR (n=1), 43% SD (n=6), 50% PD (n=7), and no patient reached a complete response.

### Ongoing and subsequent treatments

At the time of data analysis, 33% of patients (n=10) were alive, and 27% (n=8) were receiving active etiological treatment. Of these, 38% (n=3) were still receiving Avelumab, 50% (n=4) were on subsequent therapies following avelumab, and 12% (n=1) were on a subsequent therapy after pembrolizumab. Notably, no patients were still receiving pembrolizumab. In Group B, the only patient alive was receiving paclitaxel as a fourth-line treatment.

Among the four patients receiving subsequent therapies after avelumab, one was on erdafitinib for an FGFR3 S249C mutation, two were on enfortumab vedotin, and one was on paclitaxel. In the avelumab group, 69% (n=11) received a second-line therapy, distributed as enfortumab vedotin (n=5), paclitaxel (n=5), and cisplatin-gemcitabine rechallenge (n=1). Additionally, 45% (n=5) of these patients were eligible for further-line treatments.

In contrast, among the 14 patients treated with pembrolizumab in the second line, only 35% (n=5) proceeded to a third-line systemic therapy. These included enfortumab vedotin (n=1), gemcitabine (n=1), vinflunine (n=1), and vinorelbine (n=3).

In addition, local ablative radiotherapy for oligo-progressive disease during immune checkpoint inhibitor therapy was administered in 3 patients from Group A and 2 patients from Group B.

### Analysis of baseline prognostic factors

Univariable Cox regression analysis showed no significant association between NLR and survival at follow-up (HR 1.06, 95% CI 0.93-1.21, p-value 0.39). This finding was further confirmed by the analysis performed considering the dichotomized version of NLR variable (NRL ≥ 3 Vs. <3: HR 2.38, 95% CI 0.86-6.58, p-value 0.09). In both Group A and Group B, approximately 55% of patients had a baseline NLR ≥3 at the start of platinum-based chemotherapy (56% in Group A and 57% in Group B).

For what concerns PFS, no significant associations were detected with NRL (HR 1.32, 95% CI 0.95-1.83, p-value 0.10) and the dichotomized version of NRL (NRL ≥ 3 Vs. <3: HR 0.91, 95% CI 0.40-2.03, p-value 0.81).

### Adverse events

Overall, both treatments demonstrated good tolerability and safety. Severe grade 3 immune-related adverse events (irAEs) were observed in one patient receiving avelumab and three patients receiving pembrolizumab, with no Grade 4 irAEs reported (**Table 2** **).**

**Table 2:** Immuno-related adverse events.

irAEs	Avelumab (n=16)		Pembrolizumab (n=14)	
	Any grade	G≥3	Any grade	G≥3
Fatigue	5 (31%)	0	1 (7%)	0
Pruritus	2 (13%)	0	0	0
Nausea	2 (13%)	0	0	0
Dysthyroidism	2 (13%)	0	0	0
Diarrhea	1 (6%)	0	0	0
Skin rash	1 (6%)	0	1 (7%)	0
Arthromyalgias	1 (6%)	0	1 (7%)	0
Myositis	0	0	1 (7%)	1 (7%)
Infusion reaction	2 (13%)	0	0	0
Hyperglicemia	1 (6%)	1 (6%)	0	0
Acute Kidney Injury	1 (6%)	1 (6%)	0	0
Hepatitis	0	0	2 (14%)	2 (14%)

### Costs

The cost of immunotherapy was calculated for each study group. Considering the cost of each vial and the total number of cycles administered, the overall median cost of Avelumab therapy amounted to €24240, while the total cost of Pembrolizumab therapy was €11300.

## Discussion

The treatment landscape for advanced UC has evolved, with avelumab (an anti PDL-1 antibody) now established as the standard of care for maintenance therapy in mUC that remains stable or responds following first-line PBC. This recommendation is supported by the phase III JAVELIN Bladder 100 trial, which demonstrated a significant improvement in OS and PFS with avelumab maintenance therapy compared to best supportive care alone (13).

However, limited real-world data are available comparing avelumab maintenance therapy with the previous standard of care, pembrolizumab (an anti PD-1 antibody), as a second-line treatment for patients who eventually progressed after a first-line PBC (11). Retrospective comparisons of these two strategies suggest several key points. First, avelumab maintenance therapy may provide an advantage by initiating immunotherapy sooner in comparison to pembrolizumab, before progression, exploiting when patients might be more likely to tolerate treatment well and potentially benefit from prolonged disease control. Unfortunately, limited comparative data on the oncological outcomes of these treatments are available to guide clinical decision-making.

Miyake et al. reported the first real-world data in Japan comparing these two agents and second-line chemotherapy in terms of OS in mUC, and no significant difference in OS was observed when only pembrolizumab patients with SD or response to 1L chemotherapy were included in the analyses (22); Similarly, Tetsuya Shindo et al. analyzed the outcomes of pembrolizumab after platinum-based 1L chemotherapy and maintenance avelumab in patients with advanced UC using propensity score matching showing equivalent PFS and OS (23).

Real-world studies, such as the AVENANCE trial (24) suggest that patients receiving avelumab maintenance therapy may achieve longer OS particularly with extended follow-up periods. This improvement could be influenced by the introduction of novel therapies, such as antibody-drug conjugates (ADCs) like enfortumab vedotin. However, retrospective studies comparing the efficacy of enfortumab vedotin in patients with mUC treated with avelumab versus pembrolizumab have reported conflicting results (25, 26).

Moreover, direct head-to-head prospective randomized trials comparing avelumab maintenance to pembrolizumab as a second-line therapy are currently lacking (24).

In our study, the outcomes of patients treated with avelumab maintenance therapy or pembrolizumab as second-line treatment —both selected for SD or PR following PBC to ensure population homogeneity— showed similar OS and PFS (PFS from the start of Immunotherapy was 7.5 months for avelumab and 5.5 months for pembrolizumab, *p*=0.7); notably the time between the end of first-line PBC and the initiation of immunotherapy with a median interval of 4.1 months for pembrolizumab (indicating that these patients showed disease progression at the first CT scan conducted after completing chemotherapy) and 1.3 months for avelumab. It is important to highlight that the delay in immunotherapy administration may affect survival outcomes, as Pembrolizumab is administered after a disease progression, thus can influence the burden of the disease, clinical presentation and performance status of patients. Indeed, it appears that only a limited number of patients remain eligible for third-line therapy following Pembrolizumab treatment, likely due to a worsened clinical condition and increased tumor burden resulting from further disease progression.

Therefore, based on our data, the outcomes are similar, but it is true that patients treated with avelumab had a higher survival rate at data cutoff, hence the OS and PFS outcomes may improve with updated follow-up. However, it is important to consider that certain subsequent therapies, such as antibody-drug conjugates (e.g., enfortumab vedotin), were not yet approved during the time when pembrolizumab was the standard of care, which could impact the overall outcomes. Moreover, the tumor burden of patients in the II line is hypothesized to be higher with possibly less efficacy of immunotherapy strategy, which in our study has not been showing.

A further consideration is the tolerability profile of these therapies. Both avelumab and pembrolizumab are generally well tolerated, with most adverse events (AEs) being grade 1 or 2. However, avelumab maintenance therapy may expose patients to immune-related AEs over a longer period of time, as it is introduced immediately after first-line therapy rather than upon progression, which could affect patients with limited performance status. In our real-world data, treatment-related AEs were similar between the two agents.

In this context, it is also important to identify prognostic and predictive factors for therapy. As discussed in other studies (19–21), the NLR could be one factor in the context of immunotherapy. However, the analyses we conducted did not yield statistically significant results and for this reason it is not possible to determine whether this factor and the cut-off of 3 are prognostically positive or negative values.

Lastly, the cost of initiating maintenance therapy with avelumab versus delaying immunotherapy until second-line treatment with pembrolizumab is an important consideration (27, 28). Our analysis suggests that Avelumab is more expensive than pembrolizumab. This is possibly related to different factors. Indeed, the avelumab treatment is administered every two weeks until progression in an already selected responsiveness population, while pembrolizumab is given every three weeks, often without pre-medication, and after two years of treatment, can be discontinued. However, the cost-effectiveness analysis was not completed due to the small number of patients and the low response rate recorded during the immunotherapy, specifically regarding patients who received pembrolizumab (BOR during Pembrolizumab was 7% PR, 43% SD).

Additionally, we did not evaluate the indirect costs of therapies, such as monitoring or managing adverse events, which could influence the overall cost analysis. Nevertheless, considering that the incidence of irAEs was similar in our study population, we believe these factors do not significantly impact our final cost analysis.

Another important consideration arising from the results of this study is that, although the OS and PFS outcomes between Avelumab and Pembrolizumab appear comparable, it seems that few patients after pembrolizumab treatment are eligible for third-line. This could potentially affect overall survival outcomes with longer follow-up and could be related to the clinical condition of the worst tumor burden related to further disease progression.

Additionally, patient preference should be evaluated, with some possibly more afraid of experiencing disease progression after first-line treatment and others who require a treatment discontinuation after months of chemotherapy.

A key limitation of our study is the small sample size, with only 30 patients meeting the inclusion criteria. The main reason for exclusion was disease progression after four or six cycles of PBC, as we included only patients who achieved stable disease or a partial response at these time points. This selection was made to allow a meaningful comparison between patients eligible for Avelumab and those who, prior to its approval, would have received Pembrolizumab. Additionally, a significant number of patients were excluded because they were enrolled in clinical trials. Additional limitations include the retrospective study design and the relatively short follow-up period. Consequently, larger prospective studies are crucial to better understand the role of immunotherapy in mUC and to optimize treatment sequencing.

## Conclusion

Patients who did not experience disease progression after 4–6 cycles of PBC and were subsequently treated with either Avelumab as maintenance therapy or Pembrolizumab as second-line therapy showed no significant differences in OS and PFS. Notably, a higher proportion of patients remained on Avelumab treatment at the data cutoff, suggesting the potential for greater long-term benefit with Avelumab with extended follow-up. AEs were comparable between the two groups; however, cost analysis indicated that Avelumab is more expensive than Pembrolizumab.

Further insights from real-world studies and randomized head-to-head trials are crucial to defining the role of immunotherapy in mUC and optimizing treatment sequencing. These efforts should also consider cost-effectiveness and the evolving therapeutic landscape, including the emergence of promising first-line combinations (e.g., enfortumab vedotin plus pembrolizumab) (29), antibody-drug conjugates, and bispecific therapies. Such advances have the potential to further improve patients outcomes and reshape the management of metastatic UC in the near future.

## Data Availability

The raw data supporting the conclusions of this article will be made available by the authors, without undue reservation.
